# Autophagy Dances with Phytohormones upon Multiple Stresses

**DOI:** 10.3390/plants9081038

**Published:** 2020-08-15

**Authors:** Yifan Li, Yanni Lin, Xi Li, Shaoying Guo, Yifeng Huang, Qingjun Xie

**Affiliations:** 1 State Key Laboratory for Conservation and Utilization of Subtropical Agro-Bioresources, Guangdong Provincial Key Laboratory of Plant Molecular Breeding, South China Agricultural University, Guangzhou 510642, China; 20182015007@stu.scau.edu.cn (Y.L.); lyn865@yeah.net (Y.L.); 20183137023@stu.scau.edu.cn (X.L.); syguo@scau.edu.cn (S.G.); 2 Guangdong Laboratory of Lingnan Modern Agriculture, Guangzhou 510642, China; 3 Institute of Crop and Nuclear Technology Utilization, Zhejiang Academy of Agricultural Science, Hangzhou 310001, China

**Keywords:** autophagy, phytohormones, TOR, *ATG* genes, stress response

## Abstract

Autophagy is an evolutionarily conserved process for turning over unwanted cellular components, thus promoting nutrient recycling and maintaining cellular homeostasis, which eventually enables plants to survive unfavorable growth conditions. In addition to plant growth and development, previous studies have demonstrated that autophagy is involved in the responses to various environmental challenges through interplaying with multiple phytohormones, including abscisic acid (ABA), jasmonic acid (JA), and salicylic acid (SA). In this review, we summarize the advances made in their synergistic interactions in response to multiple abiotic and biotic stresses; we also discuss the remaining issues and perspectives regarding their crosstalk.

## 1. Introduction

In recent years, crop yield and productivity have been adversely challenged by the occurrence of extreme climate and environmental changes, such as drought, heat, or pathogen attack [1]. Accordingly, researchers and breeders have to develop new cultivars in order to adapt to these unfavorable conditions. Therefore, elucidating the relevant mechanisms underlying responses to environmental stresses is of particularly importance for crop genetic improvement. 

Unlike animals that can migrate to avoid stressful environments, plants have to evolve a wide variety of mechanisms in order to adapt their morphology, physiology, and metabolism to survive different biotic (including pathogen invasion and herbivores) and abiotic stresses (including nutrient deficiency, cold, heat, drought, salt stress, etc.) [2]. One of most important strategies for plants to overcome stresses is the “self-eating” regulation, which is also designated as autophagy. Autophagy is a highly conserved degradation mechanism in eukaryotes, in which the unwanted or dysfunctional cytoplasmic materials, such as proteins, protein complexes, nucleic acid aggregates, and even entire organelles, are targeted to the vacuole/lysosome for degradation [3]. Such a regulatory mechanism functions as a standby emergency supply system, providing nutrients for plants exposed to multiple environmental stresses, and then ensuring the minimum requirement for their growth and development, eventually promoting survival. 

**Figure 1:**
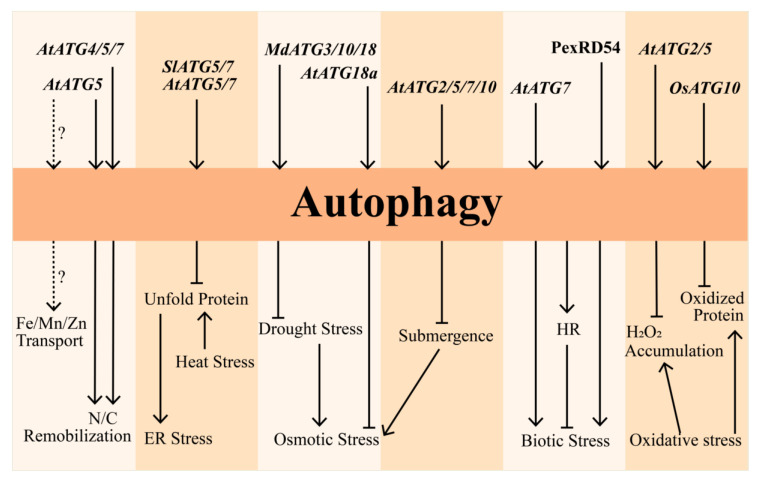
Regulation of stress response by *AuTophaGy-related* (*ATG*)-mediated autophagy. Multiple *ATG* genes operate autophagy. *AtATG5* positively regulates the nitrogen remobilization and tolerance to carbon starvation while *AtATG4* and *AtATG7* are known to modulate tolerance to carbon starvation. In addition, *AtATG5* is also involved in regulating the homeostasis of micronutrients, such as Fe/Mn/Zn. Unfold proteins were accumulated under heat stress, which has been implicated to be degraded via the *ATG5*- and *ATG7*-mediated autophagy route in Arabidopsis and tomato, eventually alleviating the unfold protein-mediated endoplasmic reticulum (ER) stress. Similarly, accumulation of H_2_O_2_ and oxidized proteins are also selectively turned over by the *AtATG2*- and *AtATG5*-dependent autophagy in Arabidopsis and the *OsATG10*-dependent autophagy in rice, respectively, ultimately relieving oxidative stress. *MdATG3*, *MdATG10*, and *MdATG18* positively enhance drought tolerance in apple, and a similar role is also found in *AtATG18a*. Furthermore, *AtATG2*, *AtATG5*, *AtATG7*, and *AtATG10* also play positive roles in regulating submergence response, finally mediating osmotic stress. Under abiotic stress, *AtATG7* positively regulates necrotrophic fungal pathogen resistance, and *pexRD54* arrests pathogen infection by boosting autophagy activity. Moreover, autophagy positively regulates hypersensitive response (HR) cell death to resist biotic stress. Solid lines represent the activation or repression with known evidence. Dashed lines represent the proposed activation or repression.

Until now, there are three distinct types of autophagy described in plants: microautophagy, macroautophagy, and mega-autophagy [4] (Figure 1). In terms of macroautophagy, cargo is trapped in the cup-shaped double membrane vesicle known as a phagophore. The phagophore then gradually expands and matures into an autophagosome, with the help of the autophagy core protein ATG8 and the Src homology-3 (SH3) domain-containing protein 2 (SH3P2) [3]. Previously, a body of studies proposed that the phagophore is formed at the endoplasmic reticulum (ER) exit sites (ERES), and the most recent evidence illustrated that it can be also generated at the ER plasma membrane contact sites (EPCS) [5,6,7]. Moreover, there is a notion that the autophagosome membrane emerges from the fused cage-like tubular network [4]. Further studies are still needed to precisely determine the origin of autophagosome. Once the autophagosome is formed, it is transported to the vacuole through the microtubule network, where its outer membrane fuses with the tonoplast to release the inner vesicle into the vacuolar lumen to form autophagic body [3,8]. This fusion step is mediated by the FYVE domain protein required for endosomal sorting 1 (FREE1) [8]. Consequently, the autophagic body is broken down into their constituent parts by a set of vacuolar hydrolases and exported back to the cytoplasm for the development of new organs or tissues. Regarding microautophagy, the tonoplast is invaginated directly to catch cytoplasmic materials that congregate at the vacuole surface to form an autophagic body, which is subsequently degraded in a similar manner as macroautophagy [9]. Collectively, both microautophagy and macroautophagy break down the autophagic body and expose the cytoplasmic materials to the vacuole for hydrolysis, finally degrading the cargo into their constituent parts for recycling to the cytoplasm [3]. Distinct from the above two types, mega-autophagy is an extreme type of autophagy, of which the tonoplast is permeabilized or ruptured to release vacuolar hydrolases directly into the cytoplasm, and then these hydrolases degrade cytoplasmic materials in the cytosol [4]. Mega-autophagy usually occurs together with programmed cell death (PCD) during the development or in response to pathogenic invasion [10]. Thus far, the molecular mechanism of autophagy is mainly focused on macroautophagy (hereafter referred to as autophagy) in plants, and little is known about microautophagy [11]. In yeast, increasing studies have demonstrated that numerous *AuTophaGy-related* (*ATG*) genes that mediate macroautophagy are also involved in microautophagy [12], but whether the plant *ATG* genes are also involved in both processes still remains elusive. 

Autophagy generally turns over proteins through the interaction with ATG8, which results in the transport of these ATG8-interacting proteins into autophagosomes. There are more than 30 ATG8-interacting proteins identified in plants (summarized in the review of Marshall et al., 2018), all of which possess the ATG8-interacting motif (AIM). Therefore, identification of AIM-containing proteins could provide broad clues for elucidating the involvement of autophagy in certain biological pathways. In general, autophagy is induced by various stresses, and thus the autophagy deficiency mutants generally display hypersensitivity to stresses [13,14,15]. In Arabidopsis, the expression of a set of *ATG* genes is significantly up-regulated in response to multiple stresses, suggesting the transcriptional regulation of autophagy by stresses [16]. However, the relevant regulatory mechanism needs to be further explored, such as how the stress signal activates *ATG* genes, which in turn initiate autophagy. 

Phytohormones play important roles in plant growth, development, and abiotic and biotic stress responses. Briefly, abscisic acid (ABA), ethylene (ET), jasmonic acid (JA), and salicylic acid (SA) were generally known to directly regulate stress responses in plants, while auxin, cytokinin (CK), gibberellin (GA), and brassinolide (BR) modulate broad physiological effects through their corresponding signaling transduction in response to stresses [8,17]. Although the crosstalk between autophagy and hormone signaling upon stress has been described recently [8,17], the detailed regulatory mechanism is still poorly understood. Genome-wide analysis of the *TaATG* promoter in bread wheat identified numerous ABA-, IAA-, GA-, ET-, MeJA-, and SA-related *cis*-elements, as well as others related to light, anoxic, heat, cold, drought, and wound stresses [18]. Similarly, another study in Arabidopsis also revealed that 225 TFs from 35 families bound to *ATG8* promoter, in which the auxin response factor (ARF), WRKY, NAC, and bZIP elements were identified. Furthermore, the TGACG (TGA) motif-binding protein 9 (TGA9) was selected as a representative for experimental validation, further indicating that this stress-related TF indeed bound to the *ATG8* promoter and then regulated the autophagy activity upon sucrose starvation and darkness treatment [16]. Taken together, these studies suggested that autophagy is modulated by a potential complicated regulation of plant hormone and stress signaling.

## 2. Regulation of Autophagy

In the past two decades, about 40 ATG components involved in autophagy machinery have been identified in yeast, and these ATG proteins exhibit a canonical route for autophagy [3], which enables the characterization of plant autophagy by genetic analysis of the homologous *ATG*s in plants (such as Arabidopsis, rice, tobacco, maize, etc.) [18,19,20,21,22]. To date, the autophagy machinery has been well-characterized among the plant kingdom, including autophagy induction; membrane delivery; vesicle nucleation; phagophore expansion and closure; and autophagosome formation and delivery, fusion, and digestion [3,23]. Accumulating evidence has elaborated the conserved role of autophagy in the growth and development among plant species, especially in Arabidopsis and rice regarding the senescence and pollen fertility [24,25]. For example, recent studies have indicated that over-expression of *ATG* genes enhance the yield and nitrogen use efficiency (NUE) in both rice and Arabidopsis [26], whereas the seed yield and nitrogen harvest index decreased in autophagy-deficient mutants due to the reduction of nitrogen remobilization for grain filling [27,28], indicating that autophagy has great potential to be of benefit in improving crop yield and productivity under either normal or suboptimal conditions. 

The target of rapamycin (TOR) complex is one of the most important negative regulators of autophagy, which is composed of TOR itself and two binding partners, the regulatory-associated protein of TOR (RAPTOR) and the lethal with Sec thirteen 8 (LST8) [29]. TOR functions upstream of autophagy in regulating plant growth and stress response [3,30,31]. Autophagy could be induced and promoted by inactivation of TOR via RNA interference (RNAi); TOR kinase inhibitors such as rapamycin and AZD8055 [32]; or disruption of its constituents, the RAPTOR and LST8 [33,34,35]. Notably, repression of autophagy by upregulation of *TOR* was specifically detected under the nutrient starvation, salt, and osmotic stresses, whereas such repression could not be identified under oxidative and ER stresses [33,36], suggesting that the TOR-mediated autophagy was only involved in the response to certain types of stresses. Previously, it has already shown that auxin functioned upstream of TOR [37]. Further analysis demonstrated that auxin also modulated autophagy in the TOR-dependent stresses response, as the exogenous application of the auxin analogue 1-naphthylacetic acid (NAA) could activate TOR to inhibit autophagy activity under salt and osmotic stresses rather than oxidative and ER stresses [36,38]. Taking into account the fact that auxin is regulated by the plant-specific family of *Rho GTPases2* (*ROP2*) [37,39], it is supposed that *ROP2* might also influence autophagy activity through the auxin–TOR pathway. Additionally, the ABA metabolism is also regulated by TOR kinase. The ABA hormone level is strongly decreased in *lst8-1* or *raptor1b* mutants, as well as in wild-type (WT) plants treated with AZD8055 [40]. These studies suggest that ABA and auxin participate in a TOR-dependent route of autophagy regulation.

The energy sensor SNF-related kinase 1 (SnRK1) is a highly conserved eukaryotic kinase protein, which responds to the nutrient and energy deficiency in plants [41]. It has been reported that sugar phosphates affect TOR activity through the modulation of SnRK1, which was shown to act upstream of TOR in Arabidopsis [42,43,44]. In particular, autophagy was inhibited by trehalose 6-phosphate (T6P), an inhibitor of SnRK1, in response to abiotic stresses [42], suggesting SnRK1 functions as an activator of autophagy. Moreover, it was also found that SNF1-RELATED PROTEIN KINASE1.1 (KIN10), a catalytic subunit of SnRK1, activates autophagy through affecting the phosphorylation of ATG1 in Arabidopsis, indicating that SnRK1 could also regulate autophagy in a TOR-independent manner [45]. This implies that *SnRK1*-triggered autophagy may be associated with TOR-independent stress responses, such as the responses to oxidative and ER stresses [42]. A recent study demonstrated that the number of autophagosomes was decreased in response to misfolded protein accumulation-induced ER stress in the *inositol-requiring enzyme 1b* (*ire1b*) mutants as compared with WT plants [46], indicating that activation of autophagy upon ER stress relies on the functional *IRE1b* in Arabidopsis. Unfortunately, the relationship between *SnRK1* and *IRE1b* in regulating TOR-independent autophagy remains unclear. Collectively, either TOR or SnRK1 functions upstream of autophagy, and then integrates autophagy and hormone signaling to regulate multiple stress responses.

## 3. Role of Autophagy under Abiotic Stress

### 3.1. Oxidative Stress

Oxidative stress is produced by free radicals, which is considered an important factor leading to aging and diseases. ROS accumulation generally causes oxidative stress, and subsequently damages most cell components, including proteins, lipids, and DNA. It has been implicated that the oxidized proteins were degraded by autophagy during oxidative stress in Arabidopsis. For example, dramatic accumulation of H_2_O_2_ could be detected in Arabidopsis *atg5* and *atg2* mutants [47,48,49,50]. Knockdown expression of *AtATG18a* in seedlings increased the sensitivity to oxidative stress as compared to WT, and other autophagy-defective plants also presented a chlorotic phenotype [50]. Furthermore, the rice *osatg10b* mutants were more sensitive to high salt and methyl viologen (MV, as an inducer of autophagy), and accumulated more oxidized proteins upon MV treatment compared with wild-type plants [14], suggesting that these oxidized proteins were selectively degraded by autophagy. Further analysis of these kinds of proteins, such as whether they could interact with ATG8 and then be transported into the vacuole for degradation, or whether over-expressing *ATG* genes could alleviate the oxidative stress, would extend our knowledge about the role of autophagy in plant responses to oxidative stress. Taken together, these studies suggest that autophagy protects plant cells from oxidative stress by degrading oxidized proteins.

### 3.2. Nutrition Starvation

An increasing number of studies have demonstrated that autophagy is involved in the recycling and remobilization of nutrients at the whole-plant level [51,52]. With respect to the nitrogen recycling, the *ATG* genes played an important role for plant survival under nitrogen-limiting conditions, since nitrogen remobilization was found to be markedly decreased in *atatg5* mutants at the vegetative stage [53,54]. Similar observations were also obtained in terms of the carbon remobilization. For instance, the *atatg4*, *atatg5*, and *atatg7* mutants displayed delayed growth under carbon starvation as compared with WT at the seedling stage [55]. Additionally, a recent study has shown that phosphate limitation could also stimulate autophagy in the root tips of Arabidopsis, and the phosphate deficiency response-2 (PDR2) and the low phosphate response-1 (LPR1) were required for the activation of autophagy under Pi starvation conditions [56]. Notably, the expression level of *ATG* genes was significantly different under carbon starvation and sucrose starvation in Arabidopsis. For instance, the *AtATG8B* was dramatically up-regulated upon sucrose starvation rather than other *ATG* genes, whereas *AtATG8A*, *AtATG8B*, and *AtATG8H* were significantly induced under carbon starvation rather than *AtATG8D*, *AtATG8E*, and *AtATG8I* [16], suggesting a distinct role of *ATG* genes in regulating these stresses. Furthermore, it is quite interesting that the transport of Fe, Mn, and Zn also required autophagy in addition to nitrogen and carbon, since an obvious decreasing of these micronutrients was identified in the seed of *atatg5* mutant. Further investigation of the double mutant of *atatg5* and *salicylic acid induction deficient 2* (*sid2)* that attenuates senescence in Arabidopsis revealed that there was a two-step mechanism underlying autophagy activity, in which the nutrients firstly were translocated into vegetative organs (such as leaf) and then remobilized to seeds [52]. Nutrition deficiency could result in early senescence of leaf, which can also be caused by darkness treatment. Taking into account that both nutrition deficiency and darkness could induce autophagy, it raises an issue as to whether these two stresses stimulate autophagy through similar or distinct mechanisms. In addition, it also needs to be explored whether autophagy would modulate nutrient transport at the EPCS, where the autophagosomes form, through affecting ion transporters. Taken together, these findings provide an insight into the fact that autophagy is essential for nutrient translocation and remobilization in plants under adverse situations.

### 3.3. Osmotic Stress

Salinity, drought, and osmotic stress caused by both these factors are the most common environmental stresses affecting plant growth and development. Knockdown of *AtATG18a* resulted in more sensitivity to salt and drought stresses as compared to WT plants [15], demonstrating that *ATG*-mediated autophagy participates in plant responses to these osmotic stresses. In accordance with the phenotype observed upon oxidative stress, the oxidized proteins in the *atatg2* and *atatg7* mutants were accumulated compared to WT plants under salt stress [57]. In addition, overexpression of *MdATG3* or *MdATG10* in apple enhances their drought tolerance [58,59]. The similar phenotype also displayed in the *MdATG18a*-overexpressing tomato [60]. Furthermore, a recent study identified a plant-specific gene, *Constitutively Stressed 1* (*COST1*), which negatively regulates drought resistance by influencing the autophagy pathway in Arabidopsis [61]. It is worth mentioning that waterlogging and submergence, as the opposite effect of drought also induces autophagosome formation in Arabidopsis. For example, the Arabidopsis *atatg2*, *atatg5*, *atatg7*, and *atatg10* mutants showed more sensitivity to submergence compared to WT plants [62], suggesting an extensive involvement of autophagy in maintaining cellular homeostasis. Notably, the morphological responses of Arabidopsis and rice to waterlogging are quite different. For Arabidopsis, the entire plant is subjected to waterlogging, while for rice the internode elongation would be triggered to escape from waterlogging. Therefore, it is interesting to characterize and compare the conserved and divergent mechanisms of autophagy in regulating waterlogging between these two species. Overall, these results illustrate that autophagy broadly regulates different types of osmotic stresses.

### 3.4. Heat and ER Stresses

High temperature is one of the major threats to plant growth and development. Heat stress causes protein misfolding and denaturation, which could also lead to ER stress [46]. Both *atatg5* and *atatg7* displayed hypersensitivity to heat stress compared to WT plants, and accumulation of insoluble protein aggregates tagged by ubiquitin was found in the *atatg7* mutant [36]. Silencing of *ATG5* or *ATG7* in tomato presents consistent response upon heat stress [63]. A recent report validated the fact that autophagy induced by heat stress actually resulted from ER stress, since the excessive unfolded proteins caused by heat were accumulated in the ER. The result was further confirmed by the sodium 4-phenylbutyrate (PBA) treatment assay, in which this chemical eliminates unfolded proteins in ER and thus reduces the heat-induced autophagy [46]. Considering the fact that autophagy turns over impaired proteins, it is strongly possible that accumulated unfolded protein would be degraded through autophagy. As expected, treatment of Arabidopsis seedlings with ER stress inducers, such as tunicamycin (TM) and dithiothreitol (DTT), precisely induced autophagosome formation [64]. Taken together, it is supposed that autophagy is responsible for the clearance of unfolded proteins existing in the ER upon heat or even other stresses.

## 4. Regulation of Autophagy under Biotic Stress

Biotic stress also influences autophagic events in addition to abiotic stress. Enhanced disease susceptibility 1 (EDS1) is a key component responsible for salicylic acid (SA)-mediated defense against diseases and pathogens [65]. Previously, it was verified that autophagy deficiency could repress the EDS1-mediated hypersensitive response (HR) cell death, a type of PCD in plants [66], suggesting a parallel role of autophagy with other PCD pathways in HR regulation. Interestingly, autophagy has been known to coordinate with SA to regulate leaf senescence [49], implying a possibility that the EDS1 or other SA regulators might be the direct targets of autophagy in response to biotic stress. Recently, other studies have further demonstrated the important role of autophagy in plant defense against pathogens, as well as the connection between autophagy and HR during plant innate immunity [42,66,67,68,69]. For example, disruption of *AtATG7* resulted in elevated susceptibility to the necrotrophic fungal pathogen, and the selective autophagy limited the cauliflower mosaic virus (CMV) infection by NBR1-mediated targeting of viral capsid protein and particles [70,71], suggesting a positive role of autophagy in biotic stress. A recent study proved that *NBR1* modulates ABA signaling in Arabidopsis [72], suggesting that autophagy-mediated CMV resistance might be also correlated with ABA signaling. Surprisingly, autophagy also restricts HR, which may be due to the different age of the plants used in the experiments, and thus a hypothesis was proposed that the role of autophagy is likely varied according to the specific pathogen [42,73,74,75]. Notably, pathogens could in turn interfere with the autophagy response from host plant. For instance, the PexRD54 from potato famine pathogen *Phytophthora infestans* physically interacted with the host ATG8CL to antagonize the binding of Joka2 and ATG8CL, which activated autophagy in host for arresting the pathogen infection [76]. Considering that SA and JA are remarkably involved in biotic stress, especially the HR and PCD regulation, it could be speculated that autophagy-mediated biotic response may be likely associated with these phytohormones.

In summary, autophagy is comprehensively involved in multiple stress responses (Figure 1). However, there are still interesting open questions regarding these regulations, such as how the *ATG* genes are activated by various stress signals and whether there is the specific role of each *ATG* gene in response to different stresses.

## 5. Autophagy Interplays with Plant Hormones upon Multiple Stresses

### 5.1. The Regulation of Autophagy by Hormones

**Figure 2:**
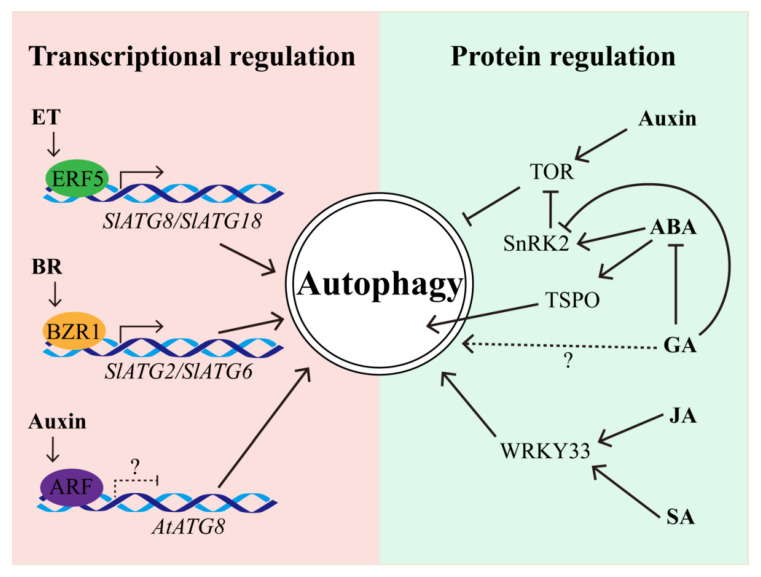
Regulation of autophagy by plant hormones. Phytohormones can modulate autophagy at the transcriptional level and through protein regulations. In respect to the former, the transcription factors ethylene response factor 5 (ERF5) in ethylene pathway and brassinazole-resistant 1 (BZR1) in the brassinolide (BR) pathway directly bind to the corresponding *ATG* promoters and then activate gene expression to promote autophagy. In contrast, the auxin regulator auxin response factor (ARF) also likely binds to the *AtATG8* promoter to repress its expression, eventually leading to attenuating autophagy activity. In addition, auxin also can repress autophagy by promoting target of rapamycin (TOR) activity. On another hand, abscisic acid (ABA) can promote the SnRK2 and TSPO to positively regulate autophagy. By contrast, gibberellin (GA) seems to antagonize ABA in regulating autophagy through inhibiting SnRK2 activity. Alternatively, GA may also directly modulate autophagy through an unknown component. Jasmonic acid (JA) and salicylic acid (SA) induce the expression of *WRKY33*, and then WRKY33 directly triggers the expression of *ATG* genes, consequently promoting autophagy. Solid lines represent the activation or repression with known evidence. Dashed lines represent the proposed activation or repression.

Autophagy could be regulated by hormones at the transcriptional level while hormones could be also modulated by autophagy in response to various stresses (Figure 2). Transcriptome analyses in petunia and Arabidopsis clearly indicated that autophagic genes could be transcriptionally regulated by hormones [77,78,79], implying phytohormones may modulate autophagy through the signaling transduction. 

ET is well known to play a dominant role in senescence and stress responses in plants. It has also been shown that ET is involved in autophagy-mediated stress response. For example, knockdown of mitochondrial alternative oxidase (AOX) decreased the level of autophagy in ET-mediated drought tolerance in tomato [79]. Inhibition of autophagy by 3-methyladenine (3-MA) resulted in decreasing disease resistance, which could be rescued by exogenous ET in banana [80]. Furthermore, autophagy genes *GmATG8i* and *GmATG4,* and ethylene biosynthesis and signaling factors *GmACS* and *GmERF* were induced in soybean under sugar or nitrogen starvation [81]. In addition, the ET signaling transcription factor, ethylene response factor 5 (ERF5), directly binds to the promoters of *SlATG8d* and *SlATG18h* and then promotes the activity of autophagy, ultimately leading to the autophagy-mediated drought tolerance in tomato [82]. Although it has been proven that the submergence-sensitive phenotype of *atatg2*, *atatg5*, *atatg7*, and *atatg10* was dependent on the *SID2*-mediated complete SA pathway, the expression of ET responsive regulators was also significantly altered in parallel with that of *ATG* genes upon submergence in Arabidopsis [62]. Therefore, it is intriguing to ask whether ET signaling-associated TF, such as ethylene insensitive-2 (EIN2) and EIN3, could also modulate the autophagy activity by regulating the expression of *ATG* genes. On the other hand, ET regulates the waterlogging through the modulation of GA-mediated elongation of internode in rice [83], implying there might be distinct crosstalk of autophagy with ET signaling between dicot and monocot plants. Taken together, these studies indicated that ET interplays with autophagy in both stress response and development in plants.

Although auxin could trigger the TOR pathway to regulate autophagy [38], it is reasonable to propose that auxin may also directly inhibit autophagy through auxin response factor (ARF) transcription factor, which may bind to the *AtATG* promoter and then modulate the gene expression (Figure 2), because the *cis*-elements of ARF have been found in the *AtATG* promoters [16]. However, this hypothesis still needs to be further explored, including the analysis of the co-expression pattern of *ARF* and *AtATGs* and the binding of ARFs with *AtATG* promoters. As an antagonist of auxin, ABA promotes bud dormancy, leaf shedding, and inhibition of cell growth. It has been mentioned that *raptor1b* mutant was unable to inhibit autophagy by exogenous NAA application [33]. Nonetheless, *raptor1b* mutant also presented a decreasing ABA level [84], suggesting that *RAPTOR1B*-mediated autophagy might be associated with ABA metabolism or signal. Furthermore, overexpression of the gene encoding heat-shock transcription factor A1a (HsfA1a) enhanced autophagy activity in tomato, whereas down-regulation of *HsfA1a* raised the plant sensitivity to ABA-mediated stomatal closure [85]. In addition, the Arabidopsis tryptophan-rich sensory protein (AtTSPO) was not only induced upon osmotic and salt stresses, but was also induced by ABA and then physically interacted with ATG8 via its AIM [86,87]. Moreover, the other two ATG-interacting proteins, ATI1 and ATI2, associated with the ER and chloroplast development, were involved in ABA-mediated seed germination [88]. Notably, TOR can phosphorylate ABA receptor PYL under favorable conditions so as to prevent the activation of stress response, which also attenuated the activity of SnRK2 kinases. Conversely, the ABA-activated SnRK2 can phosphorylate RAPTOR, thus inhibiting TOR-mediated autophagy [84]. These studies revealed that crosstalk between autophagy and ABA signaling is mediated by the phosphorylation modification of corresponding regulators [17]. GA antagonistically interplays with ABA in multiple biological processes, but little has been known about this antagonism in regulating autophagy. However, it has been implicated that GA could repress the positive regulator of ABA signaling, SnRK2, in rice [89], implying that GA might be also involved in regulating autophagy through the ABA–SnRK2 signaling pathway. 

SA plays an essential role in the activation and regulation of biotic and abiotic stress responses [90]. A recent study has shown that a high level of SA caused autophagy-mediated PCD in apple, and the phenotype of accelerated PCD during senescence and immunity in *atatg5* mutant was through the SA-dependent rather than the intact JA or ET signaling pathway [49]. Similar to SA, JA also participates in response to various biotic stresses, and the JA-related *WRKY* genes have already been implicated as mediating autophagy gene expression upon heat stress and fungal pathogen infection [17]. For example, *WRKY33* was not only involved in the JA-mediated signaling pathway to regulate plant resistance to necrotrophic fungal pathogens, but also directly interacted with ATG18a to regulate autophagy in Arabidopsis [17,91], suggesting *WRKY33* coordinates the JA signaling and autophagy pathway to regulate the pathogen stress. 

Additionally, the BR-activated transcription factor brassinazole-resistant 1 (BZR1), functioning as a positive regulator of the BR signaling pathway, was found to also directly bind to the promoters of *SlATG2* and *SlATG6* in tomato [92]. Considering the homologous conservation of *BZR1* in plants, such as Arabidopsis and rice, it is supposed that BR could also regulate autophagy through *BZR1*-dependent regulation of *ATG* expression. 

Actually, the crosstalk between autophagy and various phytohormone signaling is indeed complicated. For example, a study has been demonstrated that the decreased resistance of banana to Fusarium wilt by inhibiting autophagy could be rescued by exogenous ET, SA, and JA [80], suggesting autophagy might also in turn modulate the homeostasis of phytohormones. This topic is further discussed below.

### 5.2. Autophagy Regulates Hormone Biosynthesis and Signaling

**Figure 3:**
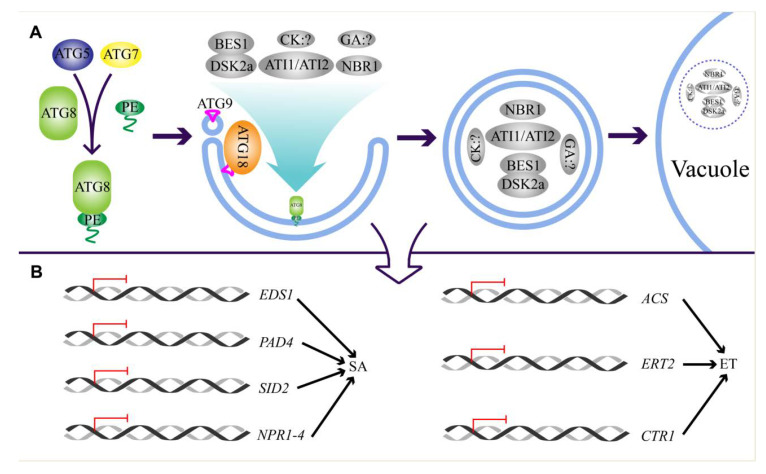
Regulation of phytohormone biosynthesis and signaling by autophagy. (**A**) Selective autophagy of phytohormone-related proteins. In the autophagic machinery, ATG5 and ATG7 are responsible for the conjugation of ATG8 to phosphatidylethanolamine (PE), which is essential for the formation of ATG8-mediated phagophore. Subsequently, the expansion of phagophore membrane and vesicle nucleation is modulated by the ATG9, which is accompanied by its interaction with ATG18, finally leading to the generation of entire autophagosome. The phytohormone-related proteins, including BRI1-EMS-suppressor 1 (BES1) and dominant suppressor of KAR 2a (DSK2a) relevant to brassinolide (BR), ATI1/2, and NBR1 relevant to ABA, and unknown components from cytokinin (CK) and GA, are transported into autophagosome by interacting with ATG8, eventually being degraded in the vacuole. (**B**) Regulation of phytohormones homeostasis by autophagy. The activation of autophagy (**A**) results in the suppression of phytohormones genes, such as the *enhanced disease susceptibility 1* (*EDS1*), *PAD4*, *salicylic acid induction deficient 2* (*SID2*) and *nonexpresser of PR genes* (*NPRI*)*1*-*4* that are involved in regulating SA signaling, and *ACS*, *ERT2* and *constitutive triple response 1* (*CTR1*) that are involved in regulating ET biosynthesis and signaling.

Apart from being regulated by hormones, autophagy can also influence hormone biosynthesis and signaling (Figure 3). For instance, a previous study has indicated that SA is accumulated in the *atatg5* mutant [49]. Furthermore, two further studies revealed that the SA signaling genes, including *EDS1*, *PAD4*, *SID2*, and nonexpresser of PR genes (*NPR*) *1-4*, and the ET biosynthetic and signaling genes, including *AtACS2*, *ethylene response 2* (*ETR2*), and *constitutive triple response 1* (*CTR1*), were upregulated in *atatg5 and atatg9* mutants upon energy starvation [79]. It is worthwhile mentioning that the soybean EIN3 accumulated upon starvation stress [82], implying that autophagy might be involved in the ET signaling transduction through selective degradation of this protein. Overexpression of *MdATG18a* resulted in less H_2_O_2_ but more SA, thereby enhancing the resistance to *Diplocarpon mali* infection [93]. In addition to SA, it has also revealed that the endogenous CK and GA concentration was significantly reduced in *osatg7* mutant in rice, and exogenous application of GA partially restored the pollen defect of *osatg7* [94]. In contrast to the Arabidopsis *atatg5* and *atatg7*, the contents of SA, JA, and ABA were not obviously altered in the *osatg7*, which might be due to different tissues used in the experiments or there is indeed a distinct role of autophagy in regulating phytohormones in rice. To address this issue, the metabolic profiling of Arabidopsis pollen and rice leaf are needed. In addition, the selective autophagy also regulated BR signaling through degrading the transcription factor BRI1-EMS-suppressor 1 (BES1) through the interaction of ATG8 with dominant suppressor of KAR 2 (DSK2) that binds BES1 [95]. Nevertheless, the interaction of NBR1 with ABA regulator protein suggests that *NBR1* also plays a role in ABA signaling by modulating its homeostasis or activity. On the other hand, NBR1 directly interacts with ATG8, further suggesting an autophagic route-dependent role of *NBR1* in fine-tuning ABA-mediated drought tolerance [13,72]. Collectively, these studies demonstrated that autophagy modulates the dynamic homeostasis of phytohormone level in addition to the signaling transduction. However, whether autophagy directly controls the turnover of the phytohormone biosynthetic proteins or the compounds remains elusive.

## 6. Future Perspectives

A steadily growing amount of evidence has revealed the essential role of autophagy in plant growth, development, and stress adaptation [36,42,48]. However, the synergistic interaction between autophagy and phytohormones upon various stresses still remains to be further exploited. Moreover, the specific regulators underlying their crosstalk needs to be identified and fully characterized. More than 20 selective autophagy receptors have been characterized from various plants, which contain the specific Atg8-interacting motif (AIM) for direct interaction with ATG8 [3,96]. The AIM generally consists of [WFY]-X-X-[LIV] consensus sequences, in which the W site is restricted by either W, F, or Y amino acid, and the L site is either L, I, or V, whereas the X represents any amino acid. Recently, two bioinformatic analyses rephrased the regular pattern of the consensus of AIM. One regular expression pattern of AIM is the [ADEFGLPRSK] [DEGMSTV] [WFY] [DEILQTV] [ADEFHIKLMPSTV] [ILV] that is termed the extended LC3-interacting region (LIR) motif (xLIR-motif) [96], while the other is composed of five regular patterns with the preference of acidic amino acids surrounding the W and L site [97]. Therefore, identification of AIM-containing proteins by bioinformatic analysis of these consensus sequences may provide a cue for autophagy-targeted, phytohormone-related proteins in addition to the traditional yeast two-hybrid screening. Additionally, a recent study revealed that the ubiquitin-interacting motif (UIM) could also interact with ATG8 [98], providing a wider range of target protein candidates that may be phytohormone- or stress-related. 

Recent studies have elaborated that overexpressing either *OsATG8a* or *OsATG8c* positively enhanced the yield and NUE in rice and the ectopic expression of *OsATG8b* in Arabidopsis also increased the yield and NUE [26,99,100], indicating that autophagy possesses great potential for boosting agronomic productivity under suboptimal condition in crops. However, whether such autophagy-mediated regulation is related with phytohormones is still poorly understood. Given the role of autophagy in turning over proteins, it may be quite interesting to explore whether phytohormone-related proteins that mediate yield and productivity are autophagy substrates. On the other hand, given that autophagy positively regulates stress response, it is worthwhile to decipher how autophagy coordinates phytohormones to maintain the cellular homeostasis under normal conditions and how it triggers the stress response upon unfavorable conditions. In summary, further exploring the regulatory network between autophagy and phytohormone signaling would extend our understanding about the stress regulation and consequently provide some new strategies and candidates for crop improvement, eventually promoting both stress resistance and yield.

## References

[1] Leng G.Y., Hall J. (2019). Crop yield sensitivity of global major agricultural countries to droughts and the projected changes in the future. Sci. Total Environ..

[2] Zhu J.K. (2016). Abiotic Stress signaling and responses in plants. Cell.

[3] Marshall R.S., Vierstra R.D. (2018). Autophagy: The master of bulk and selective recycling. Annu. Rev. Plant Biol..

[4] Van Doorn W.G., Papini A. (2013). Ultrastructure of autophagy in plant cells: A review. Autophagy.

[5] Wang P., Pleskot R., Zang J., Winkler J., Wang J., Yperman K., Zhang T., Wang K., Gong J., Guan Y. (2019). Plant AtEH/Pan1 proteins drive autophagosome formation at ER-PM contact sites with actin and endocytic machinery. Nat. Commun..

[6] Zhuang X., Wang H., Lam S.K., Gao C., Wang X., Cai Y., Jiang L. (2013). A BAR-domain protein SH3P2, which binds to phosphatidylinositol 3-phosphate and ATG8, regulates autophagosome formation in Arabidopsis. Plant Cell.

[7] Le Bars R., Marion J., Le Borgne R., Satiat-Jeunemaitre B., Bianchi M.W. (2014). ATG5 defines a phagophore domain connected to the endoplasmic reticulum during autophagosome formation in plants. Nat. Commun..

[8] Gou W., Li X., Guo S., Liu Y., Li F., Xie Q. (2019). Autophagy in plant: A new orchestrator in the regulation of the phytohormones homeostasis. Int. J. Mol. Sci..

[9] Sienko K., Poormassalehgoo A., Yamada K., Goto-Yamada S. (2020). Microautophagy in plants: Consideration of its molecular mechanism. Cells.

[10] Galluzzi L., Baehrecke E.H., Ballabio A., Boya P., Bravo-San Pedro J.M., Cecconi F., Choi A.M., Chu C.T., Codogno P., Colombo M.I. (2017). Molecular definitions of autophagy and related processes. Eur. Mol. Biol. Organ. J..

[11] Chanoca A., Kovinich N., Burkel B., Stecha S., Bohorquez-Restrepo A., Ueda T., Eliceiri K.W., Grotewold E., Otegui M.S. (2015). Anthocyanin vacuolar inclusions form by a microautophagy mechanism. Plant Cell.

[12] Ohsumi Y. (2001). Molecular dissection of autophagy: Two ubiquitin-like systems. Nat. Rev. Mol. Cell Biol..

[13] Zhou J., Wang J., Cheng Y., Chi Y.J., Fan B., Yu J.Q., Chen Z. (2013). NBR1-mediated selective autophagy targets insoluble ubiquitinated protein aggregates in plant stress responses. PLoS Genet.

[14] Shin J.H., Yoshimoto K., Ohsumi Y., Jeon J.S., An G. (2009). OsATG10b, an autophagosome component, is needed for cell survival against oxidative stresses in rice. Mol. Cells.

[15] Liu Y., Xiong Y., Bassham D.C. (2009). Autophagy is required for tolerance of drought and salt stress in plants. Autophagy.

[16] Wang P., Nolan T.M., Yin Y., Bassham D.C. (2020). Identification of transcription factors that regulate ATG8 expression and autophagy in Arabidopsis. Autophagy.

[17] Liao C.Y., Bassham D.C. (2020). Combating stress: The interplay between hormone signaling and autophagy in plants. J. Exp. Bot..

[18] Yue W., Nie X., Cui L., Zhi Y., Zhang T., Du X., Song W. (2018). Genome-wide sequence and expressional analysis of autophagy Gene family in bread wheat (*Triticum aestivum* L.). Plant Physiol..

[19] Xia K., Liu T., Ouyang J., Wang R., Fan T., Zhang M. (2011). Genome-wide identification, classification and expression analysis of autophagy-associated gene homologues in rice (*Oryza sativa* L.). DNA Res..

[20] Zhou X.M., Zhao P., Wang W., Zou J., Cheng T.H., Peng X.B., Sun M.X. (2015). A comprehensive, genome-wide analysis of autophagy-related genes identified in tobacco suggests a central role of autophagy in plant response to various environmental cues. DNA Res..

[21] Chung T., Suttangkakul A., Vierstra R.D. (2009). The ATG autophagic conjugation system in maize: ATG transcripts and abundance of the ATG8-lipid adduct are regulated by development and nutrient availability. Plant Physiol..

[22] Pei D., Zhang W., Sun H., Wei X., Yue J., Wang H. (2014). Identification of autophagy-related genes ATG4 and ATG8 from wheat (*Triticum aestivum* L.) and profiling of their expression patterns responding to biotic and abiotic stresses. Plant Cell Rep..

[23] Yoshimoto K., Ohsumi Y. (2018). Unveiling the molecular mechanisms of plant autophagy-from autophagosomes to vacuoles in plants. Plant Cell Physiol..

[24] Hanaoka H., Noda T., Shirano Y., Kato T., Hayashi H., Shibata D., Tabata S., Ohsumi Y. (2002). Leaf senescence and starvation-induced chlorosis are accelerated by the disruption of an Arabidopsis autophagy gene. Plant Physiol..

[25] Kurusu T., Koyano T., Hanamata S., Kubo T., Noguchi Y., Yagi C., Nagata N., Yamamoto T., Ohnishi T., Okazaki Y. (2014). OsATG7 is required for autophagy-dependent lipid metabolism in rice postmeiotic anther development. Autophagy.

[26] Yu J., Zhen X., Li X., Li N., Xu F. (2019). Increased autophagy of rice can increase yield and nitrogen use efficiency (NUE). Front. Plant Sci..

[27] Li F., Chung T., Pennington J.G., Federico M.L., Kaeppler H.F., Kaeppler S.M., Otegui M.S., Vierstra R.D. (2015). Autophagic recycling plays a central role in maize nitrogen remobilization. Plant Cell.

[28] Guiboileau A., Yoshimoto K., Soulay F., Bataille M.P., Avice J.C., Masclaux-Daubresse C. (2012). Autophagy machinery controls nitrogen remobilization at the whole-plant level under both limiting and ample nitrate conditions in Arabidopsis. New Phytol..

[29] Dobrenel T., Caldana C., Hanson J., Robaglia C., Vincentz M., Veit B., Meyer C. (2016). TOR Signaling and Nutrient Sensing. Annu. Rev. Plant Biol..

[30] Zhu T., Li L., Feng L., Mo H., Ren M. (2020). Target of rapamycin regulates genome methylation reprogramming to control plant growth in Arabidopsis. Front. Genet..

[31] Fu L., Wang P., Xiong Y. (2020). Target of rapamycin signaling in plant stress responses. Plant Physiol..

[32] Montane M.H., Menand B. (2013). ATP-competitive mTOR kinase inhibitors delay plant growth by triggering early differentiation of meristematic cells but no developmental patterning change. J. Exp. Bot..

[33] Pu Y., Luo X., Bassham D.C. (2017). TOR-dependent and-independent pathways regulate autophagy in *Arabidopsis thaliana*. Front. Plant. Sci..

[34] Liu Y., Bassham D.C. (2010). TOR is a negative regulator of autophagy in *Arabidopsis thaliana*. PLoS ONE.

[35] Dong P., Xiong F., Que Y., Wang K., Yu L., Li Z., Ren M. (2015). Expression profiling and functional analysis reveals that TOR is a key player in regulating photosynthesis and phytohormone signaling pathways in Arabidopsis. Front. Plant Sci..

[36] Avin-Wittenberg T. (2019). Autophagy and its role in plant abiotic stress management. Plant Cell Environ..

[37] Li X., Cai W., Liu Y., Li H., Fu L., Liu Z., Xu L., Liu H., Xu T., Xiong Y. (2017). Differential TOR activation and cell proliferation in Arabidopsis root and shoot apexes. Proc. Natl. Acad. Sci. USA.

[38] Schepetilnikov M., Dimitrova M., Mancera-Martinez E., Geldreich A., Keller M., Ryabova L.A. (2013). TOR and S6K1 promote translation reinitiation of uORF-containing mRNAs via phosphorylation of eIF3h. Eur. Mol. Biol. Organ. J..

[39] Schepetilnikov M., Makarian J., Srour O., Geldreich A., Yang Z., Chicher J., Hammann P., Ryabova L.A. (2017). GTPase ROP2 binds and promotes activation of target of rapamycin, TOR, in response to auxin. Eur. Mol. Biol. Organ. J..

[40] Kravchenko A., Citerne S., Jehanno I., Bersimbaev R.I., Veit B., Meyer C., Leprince A.S. (2015). Mutations in the Arabidopsis Lst8 and Raptor genes encoding partners of the TOR complex, or inhibition of TOR activity decrease abscisic acid (ABA) synthesis. Biochem. Biophys. Res. Commun..

[41] Crozet P., Margalha L., Confraria A., Rodrigues A., Martinho C., Adamo M., Elias C.A., Baena-Gonzalez E. (2014). Mechanisms of regulation of SNF1/AMPK/SnRK1 protein kinases. Front. Plant. Sci..

[42] Signorelli S., Tarkowski L.P., Van den Ende W., Bassham D.C. (2019). Linking autophagy to abiotic and biotic stress responses. Trends Plant Sci..

[43] Lastdrager J., Hanson J., Smeekens S. (2014). Sugar signals and the control of plant growth and development. J. Exp. Bot..

[44] Tsai A.Y., Gazzarrini S. (2014). Trehalose-6-phosphate and SnRK1 kinases in plant development and signaling: The emerging picture. Front. Plant Sci..

[45] Chen L., Su Z.Z., Huang L., Xia F.N., Qi H., Xie L.J., Xiao S., Chen Q.F. (2017). The AMP-activated protein kinase KIN10 is involved in the regulation of autophagy in Arabidopsis. Front. Plant Sci..

[46] Yang X., Srivastava R., Howell S.H., Bassham D.C. (2016). Activation of autophagy by unfolded proteins during endoplasmic reticulum stress. Plant J..

[47] Wang P., Sun X., Yue Z., Liang D., Wang N., Ma F. (2014). Isolation and characterization of MdATG18a, a WD40-repeat AuTophaGy-related gene responsive to leaf senescence and abiotic stress in Malus. Sci. Hortic..

[48] Han S., Yu B., Wang Y., Liu Y. (2011). Role of plant autophagy in stress response. Protein Cell.

[49] Yoshimoto K., Jikumaru Y., Kamiya Y., Kusano M., Consonni C., Panstruga R., Ohsumi Y., Shirasu K. (2009). Autophagy negatively regulates cell death by controlling NPR1-dependent salicylic acid signaling during senescence and the innate immune response in Arabidopsis. Plant Cell.

[50] Xiong Y., Contento A.L., Nguyen P.Q., Bassham D.C. (2007). Degradation of oxidized proteins by autophagy during oxidative stress in Arabidopsis. Plant Physiol..

[51] Masclaux-Daubresse C., Chen Q., Have M. (2017). Regulation of nutrient recycling via autophagy. Curr. Opin. Plant Biol..

[52] Chen Q., Shinozaki D., Luo J., Pottier M., Have M., Marmagne A., Reisdorf-Cren M., Chardon F., Thomine S., Yoshimoto K. (2019). Autophagy and nutrients management in plants. Cells.

[53] Thompson A.R., Doelling J.H., Suttangkakul A., Vierstra R.D. (2005). Autophagic nutrient recycling in Arabidopsis directed by the ATG8 and ATG12 conjugation pathways. Plant Physiol..

[54] Chung T., Phillips A.R., Vierstra R.D. (2010). ATG8 lipidation and ATG8-mediated autophagy in Arabidopsis require ATG12 expressed from the differentially controlled ATG12A AND ATG12B loci. Plant J..

[55] Avin-Wittenberg T., Bajdzienko K., Wittenberg G., Alseekh S., Tohge T., Bock R., Giavalisco P., Fernie A.R. (2015). Global analysis of the role of autophagy in cellular metabolism and energy homeostasis in Arabidopsis seedlings under carbon starvation. Plant Cell.

[56] Naumann C., Muller J., Sakhonwasee S., Wieghaus A., Hause G., Heisters M., Burstenbinder K., Abel S. (2019). The local phosphate deficiency response activates endoplasmic reticulum stress-dependent autophagy. Plant Physiol..

[57] Luo L., Zhang P., Zhu R., Fu J., Su J., Zheng J., Wang Z., Wang D., Gong Q. (2017). Autophagy is rapidly induced by salt stress and is required for salt tolerance in Arabidopsis. Front. Plant Sci..

[58] Wang P., Sun X., Jia X., Ma F. (2017). Apple autophagy-related protein MdATG3s afford tolerance to multiple abiotic stresses. Plant Sci..

[59] Huo L., Guo Z., Jia X., Sun X., Wang P., Gong X., Ma F. (2020). Increased autophagic activity in roots caused by overexpression of the autophagy-related gene MdATG10 in apple enhances salt tolerance. Plant Sci..

[60] Sun X., Wang P., Jia X., Huo L., Che R., Ma F. (2018). Improvement of drought tolerance by overexpressing MdATG18a is mediated by modified antioxidant system and activated autophagy in transgenic apple. Plant Biotechnol. J.

[61] Bao Y., Song W.M., Wang P., Yu X., Li B., Jiang C., Shiu S.H., Zhang H., Bassham D.C. (2020). COST1 regulates autophagy to control plant drought tolerance. Proc. Natl. Acad. Sci. USA.

[62] Chen L., Liao B., Qi H., Xie L.J., Huang L., Tan W.J., Zhai N., Yuan L.B., Zhou Y., Yu L.J. (2015). Autophagy contributes to regulation of the hypoxia response during submergence in Arabidopsis thaliana. Autophagy.

[63] Zhou J., Wang J., Yu J.Q., Chen Z. (2014). Role and regulation of autophagy in heat stress responses of tomato plants. Front. Plant Sci..

[64] Liu Y., Burgos J.S., Deng Y., Srivastava R., Howell S.H., Bassham D.C. (2012). Degradation of the endoplasmic reticulum by autophagy during endoplasmic reticulum stress in Arabidopsis. Plant Cell.

[65] Cui H., Gobbato E., Kracher B., Qiu J., Bautor J., Parker J.E. (2017). A core function of EDS1 with PAD4 is to protect the salicylic acid defense sector in Arabidopsis immunity. New Phytol..

[66] Hofius D., Schultz-Larsen T., Joensen J., Tsitsigiannis D.I., Petersen N.H., Mattsson O., Jorgensen L.B., Jones J.D., Mundy J., Petersen M. (2009). Autophagic components contribute to hypersensitive cell death in Arabidopsis. Cell.

[67] Haxim Y., Ismayil A., Jia Q., Wang Y., Zheng X., Chen T., Qian L., Liu N., Wang Y., Han S. (2017). Autophagy functions as an antiviral mechanism against geminiviruses in plants. Elife.

[68] Leary A.Y., Sanguankiattichai N., Duggan C., Tumtas Y., Pandey P., Segretin M.E., Salguero Linares J., Savage Z.D., Yow R.J., Bozkurt T.O. (2018). Modulation of plant autophagy during pathogen attack. J. Exp. Bot..

[69] Patel S., Dinesh-Kumar S.P. (2008). Arabidopsis ATG6 is required to limit the pathogen-associated cell death response. Autophagy.

[70] Lenz H.D., Vierstra R.D., Nurnberger T., Gust A.A. (2011). ATG7 contributes to plant basal immunity towards fungal infection. Plant Signal. Behav..

[71] Hafren A., Macia J.L., Love A.J., Milner J.J., Drucker M., Hofius D. (2017). Selective autophagy limits cauliflower mosaic virus infection by NBR1-mediated targeting of viral capsid protein and particles. Proc. Natl. Acad. Sci. USA.

[72] Tarnowski L., Rodriguez M.C., Brzywczy J., Piecho-Kabacik M., Krckova Z., Martinec J., Wawrzynska A., Sirko A. (2020). A selective autophagy cargo receptor NBR1 modulates abscisic acid signalling in *Arabidopsis thaliana*. Sci. Rep..

[73] Coll N.S., Smidler A., Puigvert M., Popa C., Valls M., Dangl J.L. (2014). The plant metacaspase AtMC1 in pathogen-triggered programmed cell death and aging: Functional linkage with autophagy. Cell Death Differ..

[74] Han S., Wang Y., Zheng X., Jia Q., Zhao J., Bai F., Hong Y., Liu Y. (2015). Cytoplastic glyceraldehyde-3-phosphate dehydrogenases interact with ATG3 to negatively regulate autophagy and immunity in *Nicotiana benthamiana*. Plant Cell.

[75] Wang Y., Wu Y., Tang D. (2011). The autophagy gene, ATG18a, plays a negative role in powdery mildew resistance and mildew-induced cell death in Arabidopsis. Plant Signal. Behav..

[76] Dagdas Y.F., Belhaj K., Maqbool A., Chaparro-Garcia A., Pandey P., Petre B., Tabassum N., Cruz-Mireles N., Hughes R.K., Sklenar J. (2016). An effector of the Irish potato famine pathogen antagonizes a host autophagy cargo receptor. Elife.

[77] Quijia Pillajo J.O., Chapin L.J., Jones M.L. (2018). Senescence and abiotic stress induce expression of autophagy-related genes in Petunia. J. Am. Soc. Hortic. Sci..

[78] Shibuya K., Niki T., Ichimura K. (2013). Pollination induces autophagy in petunia petals via ethylene. J. Exp. Bot..

[79] Masclaux-Daubresse C., Clement G., Anne P., Routaboul J.M., Guiboileau A., Soulay F., Shirasu K., Yoshimoto K. (2014). Stitching together the multiple dimensions of autophagy using metabolomics and transcriptomics reveals impacts on metabolism, development and plant responses to the environment in Arabidopsis. Plant Cell.

[80] Wei Y., Liu W., Hu W., Liu G., Wu C., Liu W., Zeng H., He C., Shi H. (2017). Genome-wide analysis of autophagy-related genes in banana highlights MaATG8s in cell death and autophagy in immune response to Fusarium wilt. Plant Cell Rep..

[81] Okuda M., Nang M.P., Oshima K., Ishibashi Y., Zheng S.H., Yuasa T., Iwaya-Inoue M. (2011). The ethylene signal mediates induction of GmATG8i in soybean plants under starvation stress. Biosci. Biotechnol. Biochem..

[82] Zhu T., Zou L., Li Y., Yao X., Xu F., Deng X., Zhang D., Lin H. (2018). Mitochondrial alternative oxidase-dependent autophagy involved in ethylene-mediated drought tolerance in *Solanum lycopersicum*. Plant Biotechnol. J..

[83] Kuroh T., Nagai K., Gamuyao R., Wang D.R., Furuta T., Nakamori M., Kitaoka T., Adachi K., Minami A., Mori Y. (2018). Ethylene-gibberellin signaling underlies adaptation of rice to periodic flooding. Science.

[84] Salem M.A., Li Y., Bajdzienko K., Fisahn J., Watanabe M., Hoefgen R., Schottler M.A., Giavalisco P. (2018). RAPTOR controls developmental growth transitions by altering the hormonal and metabolic balance. Plant Physiol..

[85] Wang Y., Cai S., Yin L., Shi K., Xia X., Zhou Y., Yu J., Zhou J. (2015). Tomato HsfA1a plays a critical role in plant drought tolerance by activating ATG genes and inducing autophagy. Autophagy.

[86] Hachez C., Veljanovski V., Reinhardt H., Guillaumot D., Vanhee C., Chaumont F., Batoko H. (2014). The Arabidopsis abiotic stress-induced TSPO-related protein reduces cell-surface expression of the aquaporin PIP2;7 through protein-protein interactions and autophagic degradation. Plant Cell.

[87] Vanhee C., Zapotoczny G., Masquelier D., Ghislain M., Batoko H. (2011). The Arabidopsis multistress regulator TSPO is a heme binding membrane protein and a potential scavenger of porphyrins via an autophagy-dependent degradation mechanism. Plant Cell.

[88] Honig A., Avin-Wittenberg T., Ufaz S., Galili G. (2012). A new type of compartment, defined by plant-specific Atg8-interacting proteins, is induced upon exposure of Arabidopsis plants to carbon starvation. Plant Cell.

[89] Lin Q., Wu F., Sheng P., Zhang Z., Zhang X., Guo X., Wang J., Cheng Z., Wang J., Wang H. (2015). The SnRK2-APC/C(TE) regulatory module mediates the antagonistic action of gibberellic acid and abscisic acid pathways. Nat. Commun..

[90] Maruri-Lopez I., Aviles-Baltazar N.Y., Buchala A., Serrano M. (2019). Intra and extracellular journey of the phytohormone salicylic acid. Front. Plant. Sci..

[91] Lai Z., Wang F., Zheng Z., Fan B., Chen Z. (2011). A critical role of autophagy in plant resistance to necrotrophic fungal pathogens. Plant J..

[92] Wang Y., Cao J.J., Wang K.X., Xia X.J., Shi K., Zhou Y.H., Yu J.Q., Zhou J. (2019). BZR1 mediates brassinosteroid-induced autophagy and nitrogen starvation in tomato. Plant Physiol..

[93] Sun X., Huo L., Jia X., Che R., Gong X., Wang P., Ma F. (2018). Overexpression of MdATG18a in apple improves resistance to Diplocarpon mali infection by enhancing antioxidant activity and salicylic acid levels. Hortic. Res..

[94] Kurusu T., Koyano T., Kitahata N., Kojima M., Hanamata S., Sakakibara H., Kuchitsu K. (2017). Autophagy-mediated regulation of phytohormone metabolism during rice anther development. Plant Signal. Behav..

[95] Nolan T.M., Brennan B., Yang M., Chen J., Zhang M., Li Z., Wang X., Bassham D.C., Walley J., Yin Y. (2017). Selective autophagy of BES1 mediated by DSK2 balances plant growth and survival. Dev. Cell.

[96] Kalvari I., Tsompanis S., Mulakkal N.C., Osgood R., Johansen T., Nezis I.P., Promponas V.J. (2014). iLIR: A web resource for prediction of Atg8-family interacting proteins. Autophagy.

[97] Xie Q., Tzfadia O., Levy M., Weithorn E., Peled-Zehavi H., Van Parys T., Van de Peer Y., Galili G. (2016). hfAIM: A reliable bioinformatics approach for in silico genome-wide identification of autophagy-associated Atg8-interacting motifs in various organisms. Autophagy.

[98] Marshall R.S., Hua Z., Mali S., McLoughlin F., Vierstra R.D. (2019). ATG8-binding UIM proteins define a new class of autophagy adaptors and receptors. Cell.

[99] Zhen X., Xu F., Zhang W., Li N., Li X. (2019). Overexpression of rice gene OsATG8b confers tolerance to nitrogen starvation and increases yield and nitrogen use efficiency (NUE) in Arabidopsis. PLoS ONE.

[100] Zhen X., Li X., Yu J., Xu F. (2019). OsATG8c-mediated increased autophagy regulates the yield and nitrogen use efficiency in rice. Int. J. Mol. Sci..

